# A Review of Pterostilbene Antioxidant Activity and Disease Modification

**DOI:** 10.1155/2013/575482

**Published:** 2013-04-04

**Authors:** Denise McCormack, David McFadden

**Affiliations:** ^1^Mailman School of Public Health, Columbia University, Allan Rosenfield Building, 722 West 168th Street, New York, NY 10032, USA; ^2^Department of Surgery, University of Connecticut Health Center, 263 Farmington Avenue, Farmington, CT 06030, USA

## Abstract

Pterostilbene (trans-3,5-dimethoxy-4-hydroxystilbene) is a natural dietary compound and the primary antioxidant component of blueberries. It has increased bioavailability in comparison to other stilbene compounds, which may enhance its dietary benefit and possibly contribute to a valuable clinical effect. Multiple studies have demonstrated the antioxidant activity of pterostilbene in both *in vitro* and *in vivo* models illustrating both preventative and therapeutic benefits. The antioxidant activity of pterostilbene has been implicated in anticarcinogenesis, modulation of neurological disease, anti-inflammation, attenuation of vascular disease, and amelioration of diabetes. In this review, we explore the antioxidant properties of pterostilbene and its relationship to common disease pathways and give a summary of the clinical potential of pterostilbene in the prevention and treatment of various medical conditions.

## 1. Introduction 

**Figure 1:**
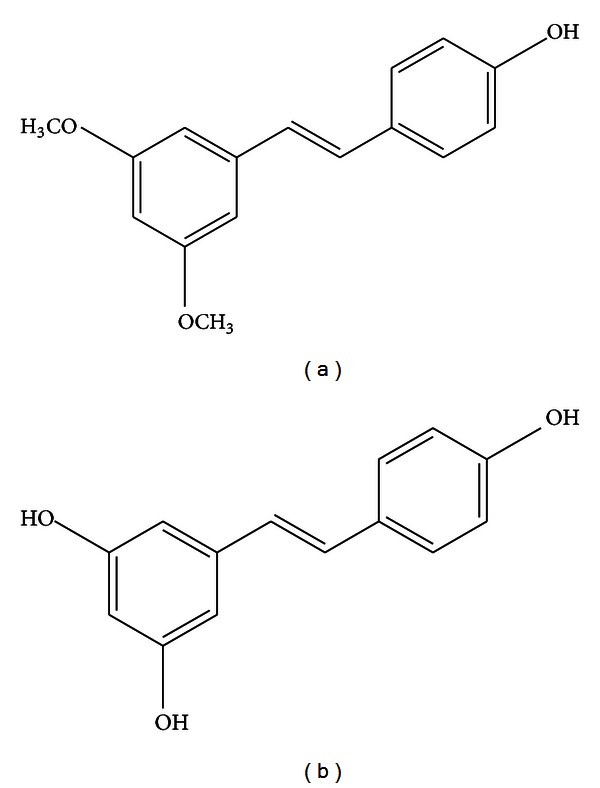
Pterostilbene (a) and Resveratrol (b). Pterostilbene contains two methoxy groups compared to Resveratrol which increases oral absorption and bioavailability.

Pterostilbene (trans-3,5-dimethoxy-4-hydroxystilbene) is a naturally derived compound found primarily in blueberries and Pterocarpus marsupium (PM) heartwood [1, 2]. The amount of daily pterostilbene consumption varies according to dietary fruit intake, and it has been estimated that pterostilbene content per blueberry varies from 99 ng to 520 ng/gram depending on the type of berry ingested [3, 4]. Substantial evidence suggests that pterostilbene may have numerous preventive and therapeutic properties in a vast range of human diseases that include neurological, cardiovascular, metabolic, and hematologic disorders. Further benefits of pterostilbene have been reported in preclinical trials, in which pterostilbene was shown to be a potent anticancer agent in several malignancies [5]. Pterostilbene is structurally similar to resveratrol, a compound found in red wine that has comparable antioxidant, anti-inflammatory, and anticarcinogenic properties; however, pterostilbene exhibits increased bioavailability due to the presence of two methoxy groups which cause it to exhibit increased lipophilic and oral absorption (Figure 1) [6–10]. In animal studies, pterostilbene was shown to have 80% bioavailability compared to 20% for resveratrol making it potentially advantageous as a therapeutic agent [6].

The multiple benefits of pterostilbene in the treatment and prevention of human disease have been attributed to its antioxidant, anti-inflammatory, and anticarcinogenic properties leading to improved function of normal cells and inhibition of malignant cells [11, 12]. Treatments with blueberry extract and PM in similar disease models have yielded paralleled results possibly due to the antioxidant activity and underlying mechanisms of pterostilbene. The evidence presented in this review shows that pterostilbene reduces oxidative stress (OS) and production of reactive oxygen species (ROS), such as hydrogen peroxide (H_2_O_2_) and superoxide anion (O_2_^−^), which are implicated in the initiation and pathogenesis of several disease processes [13]. In addition, various cell lines treated with pterostilbene have shown increased expression of the antioxidants catalase, total glutathione (GSH), glutathione peroxidase (GPx), glutathione reductase (GR), and superoxide dismutase (SOD). In this review, the clinical potential and antioxidant capabilities of pterostilbene in several disease systems will be explored and summarized. 

## 2. Antioxidant Properties of Pterostilbene 

### 2.1. Breast

Increasing rates of obesity and poor nutrition are major contributors to breast cancer occurrence in women [14]. Several studies have shown that blueberry extract and pterostilbene inhibit breast cancer *in vitro* and *in vivo*. Pterostilbene treatment of breast cancer cells has additionally been shown to alter cellular oxidative activity that may play an important role in pterostilbene-mediated cell death. 

Wu and colleagues conducted experiments to examine the effects of blueberry consumption on outcomes of early development of mammary epithelial cells in pregnant and lactating rats [15]. The authors hypothesized that blueberry consumption in pregnant rats would lead to transcriptional modification and mammary gland differentiation during the developmental period. Administering blueberry powder to pregnant and lactating rats and assessing the postnatal effects of blueberry exposure on mammary architecture and differentiation were conducted as experiments. The results of the study demonstrated that rats fed the blueberry diet exhibited higher mammary branching, increased nuclear immunoreactivity of tumor suppressor phosphatase and tensin homolog deleted in chromosome 10 (PTEN), and decreased mitotic rates. Additional experiments were performed to evaluate the effect of blueberry exposure upon *in vitro* expression of PTEN and antioxidant activity in nontumorigenic breast cells, and results revealed that cells treated with blueberry serum showed higher PTEN but similar antioxidant capacity to control cells. The findings show that breast cells are early targets of blueberry-derived mediators, which possess the ability to alter developmental mammogenesis. 

Several studies have found that blueberry exposure inhibited breast cancer *in vitro* and *in vivo* [16–18]. The results of experiments conducted by Boivin and colleagues found that blueberry juice from high-bush, low-bush, and velvetleaf blueberries exerted an antiproliferative effect against the breast cancer cell line MDA-MB-231 [17]. Likewise, Adams and colleagues found that high-bush blueberry extract decreased proliferation of triple-receptor negative breast cancer cell lines, HCC38, HCC1937, and MDA-MB-231 but did not affect proliferation of the nontumorigenic cell line, MCF-10A [18]. In the same study, treatment with blueberry extract significantly decreased human-growth-factor (HGF-) induced activation of the PI3 K/AkT/NK-*κ*B pathway, which is implicated in breast carcinogenesis. Blueberry treatment also inhibited the metastatic potential of breast cancer cells *in vitro* by inhibiting HGF-induced cell migration and matrix metalloproteinase-(MMP-) 2 and MMP-9 activity. 

In experiments utilizing a breast cancer xenograft model, treatment with blueberry extract produced smaller tumors with decreased expression of Ki-67, a marker of cell proliferation, and increased expression of caspase-3, an apoptosis marker. Blueberry fed mice also exhibited decreased activation of AkT and NK-*κ*B signaling when compared to controls [18]. The cumulative findings suggest that blueberries contain a specific chemical component capable of modifying carcinogenic pathways in breast cancer cells. 

Similarly, pterostilbene has been shown to exert anticancer effects in breast cancer through alteration of multiple cancer pathways both *in vitro *and *in vivo*. The carcinogenic pathways inhibited by pterostilbene treatment are similar to pathways altered by blueberry juice making it plausible that pterostilbene is the compound responsible for the anticarcinogenic effects of blueberry treatment in breast cancer. Moreover, pterostilbene's anticancer effects have been associated with its antioxidant-inducing capacity. Alosi and colleagues found that pterostilbene induced apoptosis and inhibited proliferation of MDA-MB-231 and MCF-7 breast cancer cells in a concentration and time-dependent manner [19]. In the same study, the antioxidant activity of pterostilbene was measured using hydroethidine (HE) to detect O_2_^−^ production. The authors hypothesized that pterostilbene when applied to cancer cells would increase O_2_^−^ production which would facilitate mitochondrial membrane depolarization triggering intrinsic mitochondriallyderived apoptosis. The results of the study show a concentration-dependent increase in O_2_^−^ production, increased mitochondrial depolarization, and associated caspase release indicating apoptosis. Mannal et al. reported that treatment with pterostilbene increased caspase release and O_2_^−^ production in breast cancer cells, consistent with the findings of Alosi [20]. 

Studies performed by Chakraborty and colleagues found that pterostilbene reduced cell proliferation and induced apoptosis through the induction of caspase-3, Bax, and p53 in breast cancer cells [21]. It was also demonstrated by Chakraborty et al. that pterostilbene treatment increased GPx antioxidant activity and the production of H_2_O_2_ and singlet oxygen indicating a mechanism of ROS-induced apoptosis [21, 22]. To evaluate the hypothesis of ROS-induced apoptosis, pterostilbene-treated breast cancer cells were treated with catalase, H_2_O_2_ scavenger, and cell survival ratios were compared to pterostilbene-treated controls. The results of the study show that catalase treatment inhibited pterostilbene-induced cell death in MCF-7 cells confirming that H_2_O_2_ is involved in pterostilbene cytotoxicity [22]. The findings of Alosi, Mannal, and Chakraborty imply that pterostilbene alters cellular oxidation to facilitate mechanisms of apoptosis in breast cancer.

The relationship between oxidation and apoptosis was further examined by Moon and colleagues who evaluated the effects of pterostilbene upon expression of Bcl-2-associated X protein (BAX), Cytochrome C, second mitochondria-derived activator of caspases (Smac/Diablo), and manganese superoxide dismutase (MnSOD), which are markers of intrinsic apoptosis [23]. The results of the study found that pterostilbene treatment produced dose-dependent increases in BAX, cytochrome C, and Smac/Diablo expression implying that pterostilbene induces intrinsic mitochondrially derived apoptosis. Pterostilbene was also shown to significantly increase MnSOD antioxidant activity in breast cancer cells, an antiproliferative mechanism that has been studied extensively [24–26]. The interconnected mechanism of increased MnSOD expression and breast cancer inhibition is not fully understood; however, it is likely that pterostilbene's ability to increase antioxidant activity is related to its function as an apoptotic and antiproliferative agent. 

Furthermore, pterostilbene treatment produced a synergistic inhibitory effect when combined with the chemotherapy drug Tamoxifen, demonstrating clinical potential in the treatment of breast cancer [20]. Further research is necessary to investigate the clinical potential of pterostilbene's antioxidant activity in human cases of breast cancer. 

### 2.2. Cardiovascular

Cardiovascular disease is currently the number one cause of mortality in the Unites States, and its high prevalence is attributed to multiple risk factors such as smoking, sedentary lifestyle and low intake of fruits and vegetables [27]. On a cellular level, vascular pathology results from dysfunction of the endothelium due to repeated exposure to mechanical stress and leukocyte generated ROS [28]. Increased mechanical stress of the endothelium predisposes to vascular injury and thrombogenicity, which is then exacerbated by increased levels of OS. The endogenous antioxidant activity of the vascular system is purported to exert a basal protective effect against pathogenesis by reducing OS; however, the antioxidant capacity of the endothelium may become exhausted from increased exposure to ROS creating an imbalance between OS and antioxidant activity [28]. Routine consumption of dietary antioxidants may therefore be protective against ROS-induced vascular injury in high-risk individuals [29]. 

Several studies have shown that blueberries, and pterostilbene alike, exhibit protective effects against cardiovascular disease possibly due to induction of antioxidant enzymes. In a study performed by Ahmet and colleagues, rats that were fed a three-month blueberry enriched diet had 22% smaller average myocardial infarction (MI) size measured 24 hours after ligation of the left descending coronary artery compared to rats fed a control diet [30]. The authors also determined that rats fed a blueberry diet had higher ejection fractions two weeks after MI compared to controls. A possible explanation for the differences in cardiac outcomes may be due to the antioxidant effects of blueberry-derived compounds leading to cardioprotection of ischemic cardiomyocytes. The theory is supported by findings that show that cardiomyocytes from blueberry fed rats exhibit a 24% increase in the ROS-induced threshold for mitochondrial permeability transition, thereby decreasing the likelihood of apoptosis in ischemic cells [30]. 

Further studies show that blueberry supplementation attenuated atherosclerosis by upregulating expression of the antioxidant enzymes SOD1, SOD2, GSR, and thioredoxin reductase- (TR-) 1 in ApoE deficient mouse models of atherosclerosis [31]. Blueberry supplementation was also shown to reduce H_2_O_2_-induced intracellular ROS production in human microvascular endothelial cells (HMVECs) [32]. 

The antioxidant activity of pterostilbene may play an important role in the cardioprotective effects observed in blueberry supplementation studies. Pterostilbene has demonstrated numerous protective benefits against atherosclerosis through regulation of vascular smooth muscle cells (VSMCs) and vascular endothelial cells (VECs). In experimental studies, VSMCs treated with pterostilbene exhibited reduction in platelet-derived-growth-factor-(PDGF-) induced proliferation and Akt, a serine-threonine kinase [33]. Pterostilbene also produced downregulation of the cell-cycle mediators, cyclin-dependent kinase (CDK)-2, CDK-4, cyclin E, cyclin D1, retinoblastoma (Rb), and proliferative cell nuclear antigen (PCNA), all of which promote unchecked VSMC proliferation resulting in atherosclerosis [33]. The results may be clinically relevant because abnormal proliferation of VSMCs is a significant component of the pathogenesis of atherosclerosis and a major contributor to the development of vascular stenosis. The antiproliferative effects of pterostilbene in VSMCs may therefore confer a defense mechanism against atherosclerosis and subsequent complications of stenosis. 

In separate studies conducted by Zhang et al., pterostilbene was shown to inhibit apoptosis and induce autophagy in VECs counteracting the proatherosclerosis effect of oxidized low-density lipoprotein (oxLDL) [34, 35]. The cytoprotective mechanism of autophagy involves removal of abnormal proteins that results from oxLDL accumulation [36]. Zhang and colleagues have demonstrated that pterostilbene treatment induces autophagy in oxLDL-stimulated VECs through activation of AMP-activated protein kinase (AMPK), intracellular calcium (Ca^2+^), and mammalian target of rapamycin (mTOR) signaling [34]. Apoptosis plays a central role in the pathogenesis of atherosclerosis through induction of plaque instability that occurs when oxLDL induces apoptosis in VECs through activation of the lectin-like oxLDL receptor −1 (LOX-1).

Activation of LOX-1 then triggers a cascade of pro-apoptosis events such as induction of p53, cytochrome C, and caspase activation. It has been postulated that increased exposure to OS facilitates apoptosis in VECs, which propagates the atherosclerosis process [37]. Treatment with pterostilbene was shown to inhibit oxLDL-induced apoptosis, suppress oxLDL-induced OS, and reduce expression of the pro-apoptosis proteins Bax and p53. Pterostilbene treatment also inhibited NF-*κ*B activation, an instrumental mediator of OS in VECs [34]. Furthermore, pterostilbene treatment suppressed oxLDL-induced expression of MMP, caspase-3/9, and attenuation of LOX-1 signaling [34]. Previous studies have shown that OS leads to LOX-1 activation that further stimulates production of OS creating a positive feedback loop that is pathogenic to vascular cells [38]. 

Pterostilbene's modulation of LOX-1, NF-*κ*B, and the antioxidant enzymes SOD and catalase indicate strong antioxidant and antiatherosclerosis effects that may be clinically significant. Although, the application of dietary pterostilbene in the prevention of cardiovascular disease is currently undetermined, a study performed by McAnulty and colleagues provided evidence that consumption of 250 g blueberry diets for three weeks attenuates angiotensin converting enzyme (ACE) activity and reduce lipid hydroperoxidase (LH), a marker of OS in chronic smokers [39]. The risk of vascular disease is high in chronic smokers, and reduction of OS by blueberry consumption may decrease the risk and/or severity of atherosclerotic disease. 

Future directions may include large-scale clinical trials to determine the impact of blueberry-derived pterostilbene in the prevention or therapy of atherosclerosis in smokers and other high-risk groups. 

### 2.3. Gastroenterology

#### 2.3.1. Esophagus

Esophageal cancer has a poor prognosis with low rates of disease survival [40]. Oxidative damage from smoking, alcohol, and gastroesophageal reflux disease (GERD) increases the risk of esophageal cancer, which some suggest may be mitigated through the use of antioxidant agents [40, 41]. In a study conducted by Stoner and colleagues, the authors evaluated the hypothesis that a blueberry diet induces anticarcinogenic, anti-inflammatory, and antioxidant effects in N-nitroso-methyl-benzylamine (NMBA) treated rats [41]. NMBA is an established carcinogen that induces tumorigenesis in the rat esophagus in a multistep fashion that is analogous to the esophageal carcinogenesis in humans. The authors found that rats treated with NMBA for five weeks, followed by a blueberry diet for a total of 35 weeks, had increased antioxidant activity, reduced tumorigenesis, and decreased expression of interleukin- (IL-) 5 and growth-related oncogene (GRO/KC), two markers of inflammation. 

Currently, the relationships between anticarcinogenesis, anti-inflammation, and increased antioxidant activity in the pathogenesis of esophageal malignancy have not been fully elucidated. 

It has been postulated that high antioxidant capacity of blueberries facilitates mechanisms of cell death in malignant cells; however, further studies are needed to assess whether the anticancer effects of blueberry treatment in esophageal cancer are related to the antioxidant effects of pterostilbene, which also induces anticancer effects in several digestive malignancies. 

#### 2.3.2. Stomach

The risk of gastric cancer is associated with genetics and dietary factors such as high consumption of smoked, salted, and nitrated foods combined with low intake of fruits and vegetables [42]. The anticancer mechanisms of fruit consumption in gastric cancer are complex; however, two studies have found that both blueberry juice and pterostilbene inhibit carcinogenesis of gastric cells. Boivin and colleagues demonstrated that juice from velvet leaf blueberry, low-bush blueberry, and high-bush blueberry, all inhibited cell proliferation of gastric adenocarcinoma cells [17]. Velvet leaf blueberry juice exerted the most significant antiproliferative effect, and high-bush blueberry juice exhibited the least significant effect. However, there was no significant correlation between antiproliferation rates and antioxidant capacity of blueberry juices leading to the conclusion that blueberries may have variable effects upon several carcinogenic mechanisms independent of antioxidant potential. Similarly, in a study performed by Pan and colleagues, pterostilbene treatment inhibited cell proliferation in a dose-dependent manner and induced apoptosis by increasing cytochrome C, Bad, Bax, and caspases 1, 2, 3, 8, and 9 in gastric adenocarcinoma cells *in vitro* [43]. 

Utilization of blueberry juice and pterostilbene to decrease gastric cancer risk and mitigate progression of malignant tumors may be a feasible option in the future. Additional research to examine the effects of blueberry juice and pterostilbene on gastric cancer should include clinical trials to assess the antioxidant and anticancer effects of blueberry-derived pterostilbene in human subjects. 

#### 2.3.3. Colon

The etiology of colon cancer is complex and involves dynamic dietary and genetic and inflammatory components [44]. Epidemiological studies have conclusively shown that chronic inflammation predisposes to high rates of colon cancer, and it has been established that diets low in fiber, fruits, and vegetables increase colon cancer risks [44]. The role of OS in the pathogenesis of colon cancer is not fully understood; however, it has been theorized that inflammation and OS interact in a positive feedback loop leading to aberrant cellular signaling and carcinogenesis. Such theories suggest that dietary antioxidants play a key role in the mitigation of colonic inflammation and colon cancer. In multiple studies, pterostilbene exhibited antioxidant properties and significantly inhibited colon cancer [45]. The anticancer effects of pterostilbene are comparable to the findings of Seeram et al. and Boivin et al. who demonstrated the inhibitory effects of blueberry compounds on colon cancer *in vitro* [16, 17]. 

Suh et al. and Chiou et al. found that pterostilbene treatment downregulated the inflammatory enzymes nitric oxide synthetase (iNOS) and cyclooxygenase-2 (COX-2), which stimulate production of proinflammatory cytokines and induce proliferation of colon cancer cells [46, 47]. Both enzymes are upregulated by OS and implicated in the progression of colonic tumorigenesis. The findings indicate that iNOS and COX-2 may be direct and indirect targets of pterostilbene's antioxidant activity in inflammation-mediated colon cancer. Chiou and colleagues also found that pterostilbene decreased expression of aldose reductase (AR), an OS protein, and increased expression of the antioxidant enzymes, GR and hemeoxygenase-1 (HO-1), through NF-E2-related factor 2(Nrf2) upregulation [47]. The transcription factor Nrf2 plays a critical role in regulation of mucosal inflammation and Nrf2 deficient mice have been shown to express increased mucosal inflammation, and OS. The results suggest that pterostilbene exerts its antioxidant effects through multiple interrelated mechanisms.

In addition to anti-inflammation and antioxidation, Chiou et al. and Suh et al. found that pterostilbene treatment inhibited colon cancer proliferation, which is consistent with the research of Remsberg et al. and Priego et al. [12, 48]. Pterostilbene treatment increased expression of the antioxidants, catalase, GPx, GR, and TR-1 by less than 2-fold and downregulated Bcl-2, a proto-oncogene implicated in colon cancer proliferation [48]. The effects of pterostilbene were most significant upon antioxidant SOD2 expression, producing a 5.7-fold increase in enzyme activity [48]. Some authors have proposed that chemotherapeutic regimens target SOD2 as an additional mechanism for tumor suppression making pterostilbene a potential chemotherapeutic agent [49]. Priego and colleagues also found that pterostilbene treatment when combined with quercetin, radiotherapy, and the chemotherapy regimen FOLFOX (oxaliplatin, leucovorin, and 5-fluorouracil) produced tumor regression in rats [48]. The overall evidence suggests that pterostilbene possesses potent anti-inflammatory, antioxidant, and anticarcinogenic properties ideal for the eradication of cancerous colon cells.

The antioxidant properties of pterostilbene may help to explain how blueberry consumption contributes to reduced risk of colon cancer in humans. Pterostilbene's modulation of antioxidant activity may also facilitate anti-inflammatory and anticarcinogenic mechanisms that confer clinical benefits in inflammatory bowel disease and colorectal malignancies. Additional studies are warranted to investigate the preventive and therapeutic effects of pterostilbene in diseases of the colon.

### 2.4. Hematology

Hemolytic disorders include a broad spectrum of hereditary and acquired conditions that range from mild to severe clinical outcomes [50]. Hemolytic anemias, irrespective of etiology, are exacerbated by exposure to ROS, which produces both internal and external damage to red blood cells (RBCs), accelerating the process of hemolysis. Studies have shown that ROS-induced hemolysis is a modifiable event that can be alleviated with antioxidant treatment [51]. Specifically, treatments with blueberry extract and pterostilbene have been shown to protect RBCs against ROS-induced hemolysis indicating a possible therapeutic effect in the treatment of hemolytic anemia. 

Youdim and colleagues conducted experiments both *in vitro* and *in vivo* to assess the antioxidant capacity of blueberry-derived polyphenolic components in vulnerable RBCs [51]. The results of experiments performed *in vitro* show that low-bush blueberry treatment reduced rates of ROS formation at 6 and 24 hours after H_2_O_2_ treatment in a time- and concentration-dependent manner. *In vivo*, oral blueberry supplementation produced significant inhibition of H_2_O_2_-induced ROS formation at 6 and 24 hours similar to the results obtained *in vitro*. Serum analysis of blueberry fed rats revealed detectable levels of anthocyanins present at 1- and 6-hour intervals but not at 24 hours, indicating a short-term protective effect. The findings of the study suggest that oral blueberry supplementation protects RBCs against ROS formation after exposure to H_2_O_2_. 

The relationship between antioxidant activity and ROS-induced RBC damage was further explored by Mikstacka and colleagues that studied the antioxidant effects of pterostilbene in RBCs that were treated with 2,2-azobis 2-amidinopropane dihydrochloride (AAPH), a known free radical generator that causes OS in RBCs leading to hemolysis [52]. The authors found that pterostilbene treatment inhibited AAPH-induced hemolysis and AAPH-induced depletion of the antioxidant enzyme GSH. Moreover, pterostilbene treatment was found to inhibit H_2_O_2_-induced lipid peroxidation, an initiator of OS that produces autoxidation in RBCs [53]. 

It has been postulated that blueberries and its component pterostilbene protect RBCs against OS by scavenging H_2_O_2_, altering the harmful effects of ROS and increasing antioxidant activity. The short-term benefits of pterostilbene are observable in RBCs up to 24 hours but long-term effects have not been studied. Currently, it is undetermined whether oral supplementation with blueberries or pterostilbene is able to prevent hemolytic episodes in humans. Future research is needed to elucidate the antioxidant enhancing mechanisms of pterostilbene and prevention of hemolysis in clinical trials. 

### 2.5. Hepatopancreaticobiliary

#### 2.5.1. Liver

Chronic liver disease (CLD) is an end-stage process that results from conditions like Wilson's disease, hemochromatosis, and primary biliary cirrhosis, in addition to infection, alcoholism, and nonalcoholic steatohepatitis (NASH) [54]. The pathogenesis of CLD is complex but follows a consistent model of progression from acute hepatic cellular injury to apoptosis, necrosis, inflammation, and irreversible fibrosis that can culminate in cancer [54]. In experimental studies, OS is a common mediator in the progression of hepatic injury to sustained inflammation and fibrosis regardless of disease etiology. For example, studies have shown that models of CLD due to viral hepatitis, NASH, alcoholism, and excess deposition of copper or iron, all share common pathways of increased OS combined with reduced antioxidant capacity [54]. An imbalance of oxidation, measured by increased ROS, H_2_O_2_, and O_2_^−  ^, can alter critical transcriptional and translational cell signaling leading to increased proliferation and eventually fibrosis of hepatic cells. The combined effect of increased OS and reduced antioxidant capacity is deleterious because it potentiates and amplifies structural damage in a time-dependent manner leading to permanent cellular and parenchymal hepatic impairment. 

Wang and colleagues administered blueberry juice to rats with CCl_4_-induced hepatic fibrosis and found an increase in the expression of the transcription factor, NF-E2-related factor 2 (Nrf-2), and its downstream target, the antioxidant enzyme NADPH quinone oxidoreductase (Nqo1), which are central to hepatic stellate cell cytoprotection [55]. Blueberry juice was shown to increase levels of the antioxidant enzymes SOD and GST and decrease malondialdehyde (MDA) levels in CCl_4_ treated mice. Levels of hyaluronic acid (HA) and alanine aminotransferase (ALT), two markers of acute hepatocyte injury, were also significantly decreased in blueberry treated rats. The study findings are consistent with results from a previous study, which found that blueberry juice increased expression of Nrf-2, Nqo1, and HO-1 [56]. Furthermore, rats treated with blueberries had increased frequency of CD3+ and CD4+ T-lymphocytes suggesting that blueberries have an immunomodulatory effect *in vivo *[56]. 

A study conducted by Osman and colleagues reported that pretreatment with blueberry powder led to decreased blood levels of bilirubin and ALT but not aspartate aminotransferase (AST) in rat models of D-galactosamine/lipopolysaccharide-induced hepatic injury [57]. Blueberry treated rats also exhibited decreased levels of proinflammatory cytokines tumor necrosis factor- (TNF-) *α* and IL-1*β* and increased levels of GSH in liver tissue, indicating combined anti-inflammatory and antioxidative effects. Additional key findings included decreased lipid peroxidation measured by MDA levels and decreased myeloperoxidase (MPO), a marker of neutrophil-derived inflammation [57]. Treatment with blueberries also inhibited proliferation of hepatic cancer cells which was demonstrated by Schmidt et al. [58]. The cumulative evidence suggests that blueberry supplementation regulates hepatic cell dysfunction through alteration of various anti-oxidative, ant-inflammatory, and antiproliferative mechanisms. 

Protection against hepatic cellular dysfunction has also been demonstrated by pterostilbene, which has been shown to thwart cellular dysfunction by inhibiting H_2_O_2_-induced inhibition of gap junctional intercellular communication (GJIC), a key facilitator of hepatic tumorigenesis [59]. Inhibition of GJIC results from activation of the extracellular receptor kinase (ERK) 1/2 and p38 pathways and phosphorylation of the gap junctional protein connexin 43 (Cx43), resulting in aberrant gap cell communication. Kim and colleagues found that pretreatment with pterostilbene at doses of 0.5, 1.0, and 5.0 *μ*M for 24 hours negated the inhibitory effects of H_2_O_2_ through dephosphorylation of Cx43 with subsequent restoration of normal GJIC [59]. Pterostilbene's antioxidant effect was found to correlate with repression of an established carcinogenic pathway, making it a potentially advantageous agent for hepatic tumor suppression.

Experiments conducted by Hasiah and colleagues found that antioxidant effects of pterostilbene were present in both cancerous and noncancerous hepatic cells [60]. Treatment with 6.25 to 100 *μ*M pterostilbene increased endogenous antioxidant activity in cancerous HepG2 hepatoma and normal Chang liver cells; however, the effect was higher in HepG2 cells. Pterostilbene also decreased cell viability of HepG2 cells that is consistent with its properties as an anticancer agent. The findings suggest that pterostilbene's antioxidant activity is beneficial to normal cells but antagonistic to the growth of cancerous cells. 

Pan and colleagues demonstrated antimetastasis effects of pterostilbene using a12-O-tetradecanoylphorbol 13-acetate- (TPA-) induced metastasis model *in vitro* and *in vivo* [61]. The results of the study found that pterostilbene treatment significantly inhibited TPA-induced vascular endothelial growth factor (VEGF), epidermal growth factor (EGF), and MMP activity *in vitro* and *in vivo,* without producing significant toxicity in rodents. The research findings demonstrate pterostilbene's potential as an antimetastasis agent, and future studies may assess whether the anti-metastatic properties of pterostilbene are applicable to human cases of hepatoma as well. 

Overall, blueberries and pterostilbene exert anti-inflammatory, antioxidant, and anticarcinogenic effects in models of CLD and liver cancer. The compound may afford clinical protection in a broad range of benign and malignant liver conditions through amelioration of OS and related hepatocyte pathology. Further research should focus upon the medicinal impact of pterostilbene in the management of CLD. 

#### 2.5.2. Pancreas

The etiology and pathogenesis of pancreatic cancer is multifactorial and involves various genetic and environmental components. It has been postulated that pancreatic cancer results from an accumulation of multiple genetic mutations making it a highly chemoresistant disease with low rates of survival [62]. Despite extensive scientific efforts, an efficacious strategy for prevention and cure of pancreatic cancer remains elusive. 

Several studies have shown that pterostilbene inhibits pancreatic cancer *in vitro* and* in vivo* through mechanisms of mitochondrially derived apoptosis, modification of transcription factors, and inhibition of proliferation [63, 64]. Specifically, pterostilbene-induced apoptosis in the pancreatic cell lines MIA PaCa-2 and PANC-1 has been attributed to mitochondrial membrane depolarization, release of Cytochrome C, and Smac/DIABLO with subsequent activation of caspase 3/7 [63, 64]. A pancreatic cancer genomic analysis of pterostilbene revealed downregulation of multiple apoptosis-related genes including MnSOD, DNA-damage-inducible transcript 3 (DDIT-3), and growth differentiation factor 15 (GDF-15), also known as macrophage inhibitory cytokine 1 (MIC-1) [64]. Further experiments demonstrated that pterostilbene induced upregulation of MnSOD at the genomic level which translated into downstream increased enzymatic activity [64]. Pterostilbene's ability to increase antioxidant activity by altering expression and enzymatic activity of MnSOD contributes to its credence as an anticancer agent because numerous studies show that pancreatic cancer cells have decreased expression of MnSOD when compared to normal cells and overexpression of MnSOD correlates with decreased pancreatic tumor volume [65–68]. In experiments conducted by Kostin et al., pterostilbene demonstrated synergistic inhibition of pancreatic cancer *in vitro* when combined with the green tea antioxidant epigallocatechin-3-gallate (EGCG) supporting previous evidence of an antioxidant effect [69]. In addition to inhibiting pancreatic cancer, recent research found that pterostilbene ameliorated inflammation and acinar damage in pancreatitis *in vitro* [70]. The collective findings indicate pterostilbene's clinical relevance in the treatment of pancreatic disease. Further studies are warranted to examine the mechanisms involved in pterostilbene-induced antioxidant activity and inhibition of pancreatitis and pancreatic cancer in clinical trials. 

### 2.6. Metabolic

Diabetes mellitus (DM) is a disease that consists of ineffective insulin regulation leading to derangements in carbohydrate, protein, and fat metabolism [71]. The disease is a component of the “metabolic syndrome,” a clinical spectrum of abnormal lipid and glucose metabolism. Over recent decades, the incidence of DM has increased worldwide due to sedentary lifestyle and the rising epidemic of obesity [72]. Lifestyle modification is one strategy employed to treat DM and associated complications; however, failure to respond to lifestyle modification is an indication for medical treatment [71]. Unfortunately, treatment with medical agents may have significant side effects, and multiple adjustments may become necessary to achieve positive clinical results. Therefore, the pursuit of new medical agents with minimal side effects remains an enviable option for the successful treatment of DM. 

The heartwood of the plant Pterocarpus marsupium (PM) has been shown to exhibit antiglycemic properties in multiple studies. In a study performed by Grover et al., rats were fed high-fructose diets to induce hyperglycemia and insulin resistance and then treated with PM orally for thirty days [73]. The authors hypothesized that PM treatment would counteract the metabolic side effects of a high-fructose diet by mitigating hyperglycemia, hyperinsulinemia and hypertriglycemia. Results of the study show that rats fed high-fructose diets combined with PM treatment had lower levels of hyperinsulinemia, hypertriglycemia, and complete prevention of hyperglycemia. It has been hypothesized that the antiglycemic properties possessed by PM are attributed to pterostilbene. Experiments performed by Manickam and colleagues assessed the antiglycemic effects of pterostilbene isolated from PM in a streptozocin- (STZ-) induced rat model of hyperglycemia and found that oral dosing of 20 mg/kg pterostilbene significantly decreased plasma glucose levels by 42% and body weight by 20% [74].

Further studies were conducted by Pari and Satheesh evaluating the antiglycemic effects of pterostilbene in combination with its antioxidant effect in rodent models of STZ-induced DM [75, 76]. The findings of the studies show that treatment with oral 40 mg/kg of pterostilbene for 6 weeks produced a significant decrease in plasma glucose levels by 56.54% and an increase in plasma insulin levels. It was also discovered that pterostilbene treatment reduced glycosylated hemoglobin (HbA1c), a marker of chronic hyperglycemia, and decreased expression of the gluconeogenic enzymes glucose-6-phosphatase and fructose-1, 6-biphosphatase. In addition, pterostilbene increased expression of the glycolytic enzyme hexokinase. The authors concluded that the effects of pterostilbene were comparable to the experimental effects of 500 mg/kg oral metformin in the STZ-induced DM model.

One proposed mechanism for the antidiabetic effects exerted by pterostilbene is reduction of OS, which plays a critical role in aberrant glucose regulation. Satheesh and Pari hypothesized that pterostilbene treatment in diabetic rats would increase antioxidant activity and lessen the impact of OS on kidney and liver cells [76]. The experimental design measured OS using thiobarbituric acid reactive substance (TBARS) and hydroperoxide (HP) levels in diabetic rats treated with 40 mg/kg oral pterostilbene in comparison to diabetic rats treated with metformin. The authors also evaluated the effect of pterostilbene upon the activity of antioxidant enzymes catalase, SOD, GPx, and GST. Results of the experiments show that DM control rats exhibited marked increases in TBARS and HP in liver and kidney tissue that was subsequently inhibited by pterostilbene treatment [76]. In DM rats, pterostilbene decreased TBARS by 61.5% and 33.3% in liver and kidney tissue, respectively. HP expression in liver and kidney was also significantly decreased by pterostilbene treatment by 27.7% and 28.3%, respectively. 

 The authors found that activity of the antioxidant enzymes GSH, GST, SOD, GPx, and catalase were decreased in liver and kidney tissue of DM controls; however, pterostilbene treated DM rats had significant increases in activity of all five antioxidant enzymes [76]. Moreover, histopathological examination of the livers of pterostilbene treated DM rats did not show inflammation compared to the DM controls, which exhibited significant portal triad inflammation. Examination of diabetic rat kidneys revealed glomeruli mesangial capillary proliferation with tubular epithelial damage that was significantly reduced in DM rats treated with pterostilbene. Comparable antioxidant and histopathological results were observed in DM rats treated with metformin suggesting that pterostilbene may harbor clinically significant metabolic properties. 

The reported antioxidant and antihyperglycemic activities of pterostilbene may confer a protective effect against complications in poorly controlled DM patients by preventing hyperglycemia and associated liver and kidney damage. The exact relationship between antioxidant activity and glucose regulation induced by pterostilbene treatment has not been elucidated; however, it is postulated that pterostilbene increases antioxidant activity leading to improved glucose metabolism. Increased antioxidant activity produced by pterostilbene may improve tissue resilience against hyperglycemia-generated ROS and prevent end-organ damage. 

The human applicability of pterostilbene's antidiabetic effects is still unknown. Nemes-Nagy et al. investigated the effect of blueberry extract on antioxidant activity in DM children and found that treatment with blueberry concentrate for two months significantly increased erythrocyte SOD and GPx activity and decreased levels of HbA1c [77]. It is possible that such results are attributable to the antioxidant activity of pterostilbene; however, additional studies are needed to identify the blueberry-derived mediator and investigate a plausible association with pterostilbene. 

In addition to mitigating hyperglycemia, pterostilbene *in vitro* and *in vivo* has shown benefits in models of lipid metabolism. In 3T3-L1 preadipocytes, pterostilbene treatment decreased cell population growth, fat droplet formation, and triacylglycerol accumulation [78]. Pterostilbene also altered gene expression of peroxisome proliferator-activated receptor- (PPAR-) *γ*, CCAAT/enhancer binding protein- (C/EBP-) *α*, resistin, and fatty acid synthase (FAS), possibly modifying early stage adipocyte differentiation and decreasing the risk of atherosclerosis [79]. Furthermore, pterostilbene demonstrated antiobesity properties by upregulating adiponectin and downregulating leptin, indicating an antilipogenic effect [79]. Expression of adiponectin negatively correlates with body mass index (BMI), glucose, insulin, and triacylglycerol levels in comparison to leptin, which positively correlates with adipocyte size, lipid content, and BMI. Rimando and colleagues found that hypercholesterolemic hamsters fed 20 ppm oral pterostilbene demonstrated a 29% decrease in plasma low density lipoprotein (LDL) cholesterol, 7% increase in high density lipoprotein (HDL) cholesterol, and 14% decrease in plasma glucose levels compared to controls [79]. The authors also found that pterostilbene *in vitro* increased PPAR-*γ* activation in rat liver cells that was significantly higher than the amount of PPAR-*γ* activation produced by the lipid-lowering agent clofibrate. Such findings are significant because derangements of glucose metabolism often accompany hyperlipidemia in diabetics and those diagnosed with metabolic syndrome. Ultimately, the glucose and lipid-lowering effects of the dietary compound pterostilbene may contribute to its clinical potential for prevention or treatment of diabetes. Further research is necessary to establish pterostilbene's risk-reducing and therapeutic effects in DM individuals. 

### 2.7. Neurology

The aging process in humans is associated with acquired deficiencies in cognition and motor function. The process is oftentimes innocuous; however, in certain neurological conditions such as Alzheimer's Disease (AD), the effects of aging are pathological and accelerated leading to rapid and permanent neurological decline [80]. Increased OS due to progressive declines in antioxidant activity is a proposed mechanism of age-related neurological deterioration in older adults [81, 82]. Several studies show that consumption of berries rich in antioxidants may effectively thwart neurological deterioration associated with aging [83, 84]. 

In an experiment conducted by Joseph et al., dopamine (DA) treatment induced OS in fibroblast cells transfected with the striatal muscarinic receptors (MAChr) subtypes M1 and M3 AChR by increasing activation of pCREB and pPKC*γ* [85]. The MAChr subtypes M1 and M3 AChR exhibit increased sensitivity to OS and are implicated in neurodegeneration making them reliable markers of OS-induced dysfunction. The authors found that treatment with blueberry extract decreased dopamine- (DA-) induced upregulation of the oxidative mediators, CREB and pPKC*γ*, indicating a significant antioxidant effect [85]. 

Bickford and colleagues examined the effect of blueberry supplementation upon antioxidant activity in aged rats along with corresponding neurological pathways and behavioral outcomes [86]. In the cerebellar noradrenergic system, reduced *β*-adrenergic function is associated with motor learning deficits in animal models. For example, cerebellar Purkinje cells in aged rats show a 30% response to *β*-adrenergic (GABA) potentiation, compared to 70% in young rats. Furthermore, such changes correlate with impaired performance of motor learning and coordination. Bickford and colleagues found evidence that blueberry-fed aged rats had significant improvements in GABA potentiation and increased GSH compared to aged controls. In addition, blueberry-fed aged rats performed rod-running motor tasks at a faster pace compared to controls. The reported findings show that blueberries contain a compound that is capable of increasing GSH antioxidant activity and cerebellar Purkinje cell GABA potentiation resulting in enhanced psychomotor performance in aged rats. Comparable findings were obtained by Malin and colleagues who demonstrated that aged rats maintained on a 1- or 2-month blueberry diet showed significantly higher object memory recognition compared to control rats [87]. The cognitive benefits were seen after termination of the blueberry intervention diet where the 2-month blueberry diet had a longer benefit compared to the 1-month diet suggesting a time-dependent neuroprotective benefit. 

Pathologic examination of the cerebellum, cortex, and hippocampal regions of blueberry fed rats that revealed significant expression of blueberry-derived polyphenolic compounds in regions important for learning and memory assessed the impact of blueberry supplementation on brain tissue [88]. The findings suggest that blueberry-derived compounds exert neuroprotective effects by crossing the blood brain barrier and altering central nervous system signals. The study results found that accumulation of polyphenolic compounds in the cortex correlated with Morris water maze (MWM) performance, which indicates a possible risk-reducing relationship between blueberry-derived polyphenolic compounds and memory and spatial learning abilities. Furthermore, in experiments performed by Casadesus et al., supplementation with blueberry extract was shown to enhance hippocampal plasticity and increase levels of insulin-like growth factor (IGF-) 1, IGF-2, and ERK resulting in improved spatial memory [89]. The findings are considerable because age-related memory decline has been attributed to hippocampal deficiencies that are mediated by IGF-1, IGF-2, and ERK pathways. 

The beneficial effects of blueberries in the modulation of neurological function may also be applicable to clinical conditions such as stroke and AD. In a study conducted by Sweeney et al., which examined the role of dietary blueberry extract on ischemic brain damage outcomes in rat stroke models, rats that were treated with 14.3% blueberry extract for six weeks had 17% loss of hippocampal neurons compared to 40% in control rats [90]. In experiments using the APP/PS1AD mouse model, it was determined that blueberry supplementation reversed deleterious effects of aging due to observations that blueberry fed APP/PS1 mice did not exhibit deficits in maze performance or have high amyloid beta burden compared to controls [84]. 

The neuroprotective effects of a blueberry-enriched diet are numerous, and several studies have sought to identify and explain the blueberry-derived compound responsible for the multiple modulatory effects of blueberry supplementation in animal models. To determine whether pterostilbene was involved in neuroprotective outcomes, Joseph and colleagues treated aged rats with low (0.004%) and high (0.016%) dose pterostilbene and evaluated endpoints of cognitive and motor functions [83]. The study results show that pterostilbene fed aged rats performed better on cognitive and motor tasks compared to controls in a dose-dependent manner. Specifically, aged rats treated with pterostilbene had higher level MWM performance, which was similarly shown in a blueberry supplementation study conducted by Andres-Lacueva and colleagues [88]. The study findings suggest that pterostilbene may be involved in modulation of neural plasticity and associated cognitive and motor functions. 

Furthermore, Joseph et al. found that low and high dose pterostilbene fed rats had serum levels of 3.951 ± 0.439 ng/mL and 25.576 ± 5.411 ng/mL pterostilbene, respectively [83]. Subsequent pathological examination of hippocampal samples found detectable levels of pterostilbene in high dose fed rats but did not reveal detectable levels in low dose fed animals. Hippocampal levels of pterostilbene correlated with working memory performance that suggests that improvements in neurological function may be directly related to pterostilbene consumption.

In a study performed by Chang and colleagues, the antioxidant potential of pterostilbene was examined in the accelerated aging mouse model SAMP8 to determine a possible relationship between the antioxidant capacity of pterostilbene and neurological markers of disease [91]. SAMP8 mice exhibit increased OS, hyperphosphorylation of tau, and cognitive decline, which were ameliorated in mice that were fed 120 mg/kg pterostilbene for eight weeks. Pterostilbene fed SAMP8 mice also showed improved performance in radial arm water maze trials and significant changes in MnSOD, PPAR-*α*, phosphorylated JnK, and phosphorylated tau, all of which play an important role in the pathology of AD. 

Pterostilbene-induced upregulation of MnSOD in SAMP8 mice indicates a modifiable defense mechanism against the harmful effects of ROS associated with neurological decline. Pterostilbene was also shown to increase levels of PPAR-*α*, an upstream inducer of MnSOD, and decrease levels of phosphorylated JnK and tau, both of which are associated with OS signaling dysfunction [91]. Pterostilbene-induced upregulation of PPAR-*α* may have potential clinical benefits since PPAR agonists have been shown to confer central nervous system protection and be therapeutic after stroke [96]. 

Overall, the antioxidant capacity of pterostilbene has significant effects upon neurological function that may translate into clinical benefits in human subjects. The free radical theory of aging claims that ROSs are involved in the pathogenesis of age-related neurological decline. Moreover, several studies suggest that AD results from decreased activity in major antioxidant defense systems and subsequent increased vulnerability to OS [80, 81]. Blueberry-induced increases in GSH and pterostilbene's ability to increase PPAR-*α* and MnSOD may abrogate the deleterious effects associated with aging and lead to improved cognition and motor function in older adults and those diagnosed with AD. Additional research is needed to evaluate clinical outcomes associated with pterostilbene treatment in AD and other severe forms of dementia. 

### 2.8. Prostate

Epidemiological trials have shown an association between poor diets and increased risk of prostate cancer [97]. Consumption of dietary antioxidants is thought to reduce prostate cancer risk in some men by reducing inflammation and OS [97]. Specifically, blueberry juice was shown to inhibit proliferation and regulate cell cycle dysfunction in prostate cancer cells [16, 17, 58]. Schmidt and colleagues found that blueberry anthocyanins inhibited cell growth of prostate cancer by 11% and inhibited adhesion of *Escherichia coli*, the bacteria primarily associated with urinary tract infections [58]. Matchett and colleagues discovered that blueberry treatment decreased activity of metastasis mediators MMP-2 and MMP-9 through alteration of protein kinase C (PKC) and mitogen-activated protein (MAP) kinase pathways and increased endogenous tissue inhibitors of metalloproteinases (TIMPs) [92, 93]. 

It has been postulated that the anticarcinogenic effect of blueberries in prostate cancer is predominantly a result of the anticancer mechanisms of pterostilbene. Studies show that pterostilbene treatment inhibits prostate cancer proliferation and reduces metastatic potential. In p53 wildtype prostate cancer cells, pterostilbene prevented cell cycle progression at the G1 phase by inducing p53 expression and upregulating p21 expression maintaining tight control of proliferation; however, in p53 negative PC3 cells, pterostilbene induced apoptosis [94]. Such findings may help to explain the beneficial effects of pterostilbene in normal cells in contrast to the cytotoxic effects observed in cancerous cells. 

Pterostilbene treatment also modified the antioxidant activity of prostate cancer cells suggesting a possible relationship between mechanisms of oxidation and apoptosis. Chakraborty and colleagues found that pterostilbene modified Bcl-2, Bax, and caspase 3, markers of mitochondrial apoptosis, and increased expression of the antioxidant enzymes GPx, GR, and GSH by 1.4-, 1.6-, and 2.1-fold in prostate cancer cells [21]. The same study also determined that pterostilbene increased levels of ROS by 5-fold, which is thought to play a role in the facilitation of mitochondrial depolarization leading to intrinsic apoptosis.

The findings demonstrate the antioxidant properties of pterostilbene in human prostate cancer cells through upregulation of the enzymes GPx, GR, and GSH. The paradoxical increase in ROS production in pterostilbene treated cells may occur through alteration of specific carcinogenic mutations present in prostate cancer that lead to programmed cell death. The findings indicate that pterostilbene is capable of inducing apoptosis through ROS-mediated mechanism in prostate cancer cells, despite upregulation of basal antioxidant activity. 

In a study by Wang and colleagues, pterostilbene treatment inhibited cell viability, induced cell cycle arrest at the G1/S phase, and upregulated cyclin-dependent kinase inhibitors, CDNK1A and CDNK1, in prostate cancer [95]. Pterostilbene also decreased prostate-specific antigen (PSA), a human marker of prostate malignancy, indicating potential use as a chemotherapeutic agent [95]. Currently, the preventive and chemotherapeutic potential of pterostilbene in human prostate cancer has not been established; however, the evidence suggests that pterostilbene may have alternate effects on prostate cells based upon genetic composition of each cell, becoming beneficial in the regulation of normal prostate cells and producing inhibition in cancerous cells. Further studies are warranted to investigate the relationship between the antioxidant effects of pterostilbene and clinical outcomes in prostate cancer. 

## 3. Discussion

The antioxidant activity of pterostilbene is an essential component of the compound's interrelated mechanisms of disease inhibition, and the studies presented in this review show that the mechanisms of pterostilbene are comparable to mechanisms exhibited by blueberry treatment in similar disease models (Table 1). The overlap is significant because blueberries are a widely consumed fruit comprised of various concentrations of pterostilbene with proven high antioxidant capacity [3, 4, 98]. Although it is postulated that the pterostilbene component of blueberries exerts clinical benefits, the direct correlation between pterostilbene's therapeutic effects and blueberry consumption remains undetermined.

The results presented in this review exemplify pterostilbene's complicated effect upon antioxidant activity and critical pathways of pathogenesis in multiple organ systems. The benefits of pterostilbene are vast and include neuroprotection, inhibition of malignancy, attenuation of atherosclerosis, protection against hemolysis and liver disease, and metabolic regulation of DM and hyperlipidemia. In breast, esophageal, stomach, colon, liver, pancreatic, and prostate cancer studies, pterostilbene exhibits profound anticancer mechanisms which include reduction of proliferation rates, induction of apoptosis, alteration of the cell cycle, and inhibition of metastasis [5]. The relationship between pterostilbene and oxidation in cancer cell death has not been fully elucidated; however, it has been discovered that generation of ROS plays a significant role in the apoptotic mechanism in pterostilbene treated breast and prostate cancer cells [19–22]. In contrast, treatment with pterostilbene increased antioxidant activity in esophageal, pancreatic, and colon cancer models but still exerted effective anticarcinogenic effects [41, 47, 48, 64]. The differences in pterostilbene's oxidative influences among cancer cell types may possibly be attributed to the distinctive daily functions of digestion which occur in the esophagus, pancreas, and colon but are absent in the breast and prostate.

Furthermore, numerous studies show that pterostilbene mechanisms vary in each disease system and are tailored toward the correction of aberrant cellular pathways and progressive dysfunction. In disease models of aging, vascular disease, diabetes, and hemolysis, pterostilbene decreases oxidative stress most likely as a protective measure against the progressive cellular damage and dysfunction associated with disease-related deterioration [34, 52, 75, 76, 91]. Interestingly, pterostilbene treatment may upregulate or downregulate specific pathways based upon the nature of the disease process taking place. For example, pterostilbene is efficacious as an anticancer agent because it induces apoptosis in cancer cells; however, the compound has the opposite effect in the vascular system where it inhibits apoptosis in VECs thereby decreasing the risk of plaque instability [5, 34]. Furthermore, in models of hyperlipidemia, pterostilbene increased expression of PPAR-*γ*, a target for lipid lowering agents, but exerted the opposite effect in AD models where it increased PPAR-*α*, a key modulator of neural antioxidant activity [79, 91]. 

Pterostilbene was also shown to exhibit comparable and synergistic effects when compared to medications used in the treatment of human disease, specifically clofibrate, metformin, Tamoxifen, and the chemotherapy regimen FOLFOX indicating that pterostilbene's therapeutic effects may be applicable if administered to human subjects [20, 48, 75, 79]. Additional possible human benefits of pterostilbene include reduction of the clinical markers HbA1C in diabetes and PSA in prostate cancer which was demonstrated by Pari and Satheesh and Wang et al., respectively [75, 95]. However, it is unknown if the beneficial effects of pterostilbene demonstrated *in vitro* and *in vivo *occur in humans as well. 

In a recent randomized double-blind placebo-controlled trial, Riche and colleagues report that 100 mg to 250 mg daily of pterostilbene in adults with hyperlipidemia did not produce significant adverse drug events [99]. In addition, treatment with 450 mg daily *Pterocarpus marsupium* extract in healthy volunteers did not produce signs of toxicity and resulted in detectable pterostilbene serum levels up to two weeks after administration [100]. The reported findings show that pterostilbene is safe for administration to humans and further contributes to our understanding of the clinical effects of pterostilbene. Further research should include study designs aimed to delineate pterostilbene's contribution to the antioxidant effects of blueberries in diverse preclinical and clinical disease models. Additional directions should focus upon the creation of human population studies and clinical trials to evaluate the safety and efficacy of pterostilbene in the prevention and treatment of disease.

## Figures and Tables

**Table 1:** Antioxidant and disease modification mechanisms of pterostilbene.

Cell type	Mechanism	References
Breast		
Blueberry	↑Mammary branching, ↑PTEN, ↓mitotic rate	Wu et al. [15]
	↓Proliferation	Seeram et al. [16], Boivin et al. [17]
	↓Proliferation, ↓PI3K/AkT/NK-*κ*B, ↓MMP, ↓Ki-67, ↑caspase 3	Adams et al. [18]
	↓Cell viability	Remsberg et al. [12]
Pterostilbene	↓Cell viability, ↑apoptosis, ↑mitochondrial depolarization, ↑superoxide anion, ↑caspase 3/7	Alosi et al. [19]
	↑Caspase 3/7, ↑S phase, ↑superoxide anion, ↓cell viability, ↑apoptosis Synergistic inhibition with Tamoxifen	Mannal et al. [20]
	↑Apoptosis, ↑caspase 3,** ↑**GPx, ↑Bax, ↑p53, ↓Bcl-2, ↓Akt, ↓MMP ↑Autophagy, ↓mitotic and metastatic potential	Chakraborty et al. [21, 22]
	↑Bax, ↑cytochrome C, ↑Smac/Diablo, ↑MnSOD	Moon et al. [23]

Cardiovascular		
Blueberry	↑Mitochondrial depolarization threshold, ↓myocardial infarction size ↑Ejection fraction	Ahmet et al. [30]
	↑SOD1, ↑SOD2, ↑GSR, ↑TR-1, ↓atherosclerosis lesion	Wu et al. [31]
Pterostilbene	↓ROS in HMVECs	Youdim et al. [32]
	↓Proliferation, ↓Akt, ↓CDK, ↓cyclin, ↓Rb, ↓PCNA in VSMCs	Park et al. [33]
	↓oxLDL-induced apoptosis, ↓oxidative stress, ↓MMP, ↓Caspase 3/9 ↑Autophagy, ↓LOX-1 signaling ↓NF-*κ*B, ↓Bax and p53 in VECs	Zhang et al. [34, 35]
	↓ACE activity, ↓LH in smokers	McAnulty et al. [39]

Esophagus		
Blueberry	↑Antioxidant activity, ↓IL-5, ↓GRO/KC	Stoner et al. [41]

Stomach		
Blueberry	↓Proliferation	Boivin et al. [17]
Pterostilbene	↓Proliferation, ↑apoptosis, ↑cytochrome C, ↑caspases 1, 2, 3, 8 and 9 ↓Bcl-XL, ↑Bad, ↑Bax	Pan at el. [43]

Colon		
Blueberry	↓Proliferation	Seeram et al. [16], Boivin et al. [17]
Pterostilbene	↓Cell viability	Remsberg et al. [12]
	↓Aberrant crypt foci, ↓iNOS, ↓COX-2, ↑MUC-2	Suh et al. [46]
	↓Aberrant crypt foci, ↓lymphoid nodules, ↓NF-*κ*B ↓iNOS, ↓COX-2**, ↑**HO-1, ↑GR, ↓Aldose reductase	Chiou et al. [47]
	↓Tumor volume, ↓NF-*κ*B, ↓Bcl-2, ↑Bax, ↑Bak, ↑Bad ↑Bid, ↑SOD2 ↑catalase, ↑GPx, ↑GR, ↑TR-1, synergistic inhibition with FOLFOX	Priego et al. [48]

Hematology		
Blueberry	↓ROS	Youdim et al. [51]
Pterostilbene	↓AAPH-induced hemolysis, ↓GSH depletion, ↓lipid peroxidation	Mikstacka et al. [52]

Liver		
Blueberry	**↑ **Nqo1, ↑SOD, ↑GST, ↑Nrf-2, ↓MDA, ↓HA, ↓ALT ↑Nrf-2, ↑Nqo1, ↑HO-1, ↑T-lymphocytes	Wang et al. [55, 56]
	↓ALT, ↓bilirubin, ↑GSH, ↓TNF-*α*, ↓IL-1*β*, ↓lipid peroxidation	Osman et al. [57]
	↓Proliferation of hepatic cancer cells	Schmidt et al. [58]
Pterostilbene	Protect gap junctional intercellular communication ↑Dephosphorylation of Cx43	Kim et al. [59]
	↓Proliferation of hepatic cancer cells	Remsberg et al. [12]
	↓Cell viability, **↑**antioxidant activity	Hasiah et al. [60]
	↓Micrometastasis↓PI3K, ↓Akt, ↓NF-*κ*B, ↓MMP-9, ↓VEGF, ↓EGF ↓Lung metastasis	Pan et al. [61]

Pancreas		
Pterostilbene	↓Cell viability, ↑apoptosis, ↑caspase 3/7, G0/G and S phase arrest	Mannal et al. [63]
	↓Tumor volume, ↑apoptosis gene expression, ↑cytochrome C, **↑**MnSOD ↑Smac/DIABLO, ↓JAK/STAT3, ↓lipase secretion, ↓IL-1*β*, ↓IL-6	McCormack et al. [64, 70]
	Synergistic inhibition of cancer proliferation with antioxidant EGCG	Kostin et al. [69]

Metabolic		
Diabetes		
Blueberry	↑SOD, ↑GPx, ↓HbA1c	Nemes-Nagy et al. [77]
Pterocarpus marsupium	↓Hyperinsulinemia, ↓hypertriglyemia, ↓hyperglycemia	Grover et al. [73]
Pterostilbene	↓Plasma glucose, ↓rat body weight	Manickam et al. [74]
	↑Plasma insulin, ↓plasma glucose, ↓HbA1c, ↓glucose-6-phosphatase ↓Fructose-1, 6-biphosphatase, ↑hexokinase, comparable to metformin	Pari and Satheesh [75]
	↓Oxidative stress, ↑GSH, ↑GST, ↑SOD, ↑GPx, ↑catalase ↓Portal triad inflammation, ↓renal damage	Satheesh and Pari [76]

Hyperlipidemia		
Pterostilbene	↓Preadipocyte growth, ↓fat droplet formation, ↓triacylglycerol	Hsu et al. [78]
	↑Adiponectin, ↓leptin, ↓LDL, ↑HDL, ↓PPAR-*γ*, ↓C/EBP-*α*, ↓resistin ↓FAS, comparable to clofibrate	Rimando et al. [79]

Neurology		
Blueberry	↑Maze performance, ↓amyloid beta burden, ↓CREB, ↓pPKC*γ*	Joseph et al. [84, 85]
	↑GABA potentiation, ↑GSH	Bickford et al. [86]
	↑Object memory recognition	Malin et al. [87]
	↑Maze performance	Andres-Lacueva et al. [88]
	↑Hippocampal plasticity, ↓IGF-1, ↓IGF-2, ↓ERK	Casadesus et al. [89]
Pterostilbene	↓Ischemic brain damage	Sweeney et al. [90]
	↑Maze performance, ↑cognitive performance	Joseph et al. [83]
	↑MnSOD, ↑PPAR-*α*, ↓phosphorylated JnK and tau	Chang et al. [91]

Prostate		
Blueberry	↓Cancer cell growth	Schmidt et al. [58]
	↓MMP, ↑TIMP	Matchett et al. [92, 93]
Pterostilbene	↓Cell viability	Remsberg et al. [12]
	↑Apoptosis, ↑caspase 3, **↑**GPx,** ↑**GR, ↑Bax, ↑p53, ↓Bcl-2, ↓Akt ↓MMP, ↑GSH	Chakraborty et al. [21]
	↑Apoptosis, ↑p53, ↑p21, G1 phase arrest	Lin et al. [94]
	↑Apoptosis, G1/S phase arrest, ↑CDNK1A and CDNK1B, ↓PSA	Wang et al. [95]
